# Motivational incentives and methylphenidate enhance electrophysiological correlates of error monitoring in children with attention deficit/hyperactivity disorder

**DOI:** 10.1111/jcpp.12069

**Published:** 2013-05-13

**Authors:** Madeleine J Groom, Elizabeth B Liddle, Gaia Scerif, Peter F Liddle, Martin J Batty, Mario Liotti, Chris P Hollis

**Affiliations:** 1 Division of Psychiatry, Institute of Mental Health, University of NottinghamNottingham, UK; 2 Department of Experimental Psychology, University of Oxford and St. Catherine’s CollegeOxford, UK; 3 Department of Psychology, Simon Fraser UniversityBurnaby, BC, Canada

**Keywords:** ADHD, electrophysiology, error monitoring, motivation, methylphenidate, stimulant medication

## Abstract

**Background** Children with attention deficit hyperactivity disorder (ADHD) are characterised by developmentally inappropriate levels of hyperactivity, impulsivity and/or inattention and are particularly impaired when performing tasks that require a high level of cognitive control. Methylphenidate (MPH) and motivational incentives may help improve cognitive control by enhancing the ability to monitor response accuracy and regulate performance accordingly.

**Methods** Twenty-eight children with DSM-IV ADHD (combined type) aged 9–15 years and pairwise-matched typically developing children (CTRL) performed a go/no-go task in which the incentives attached to performance on no-go trials were manipulated. The ADHD group performed the task off and on their usual dose of MPH. CTRL children performed the task twice but were never medicated. EEG data were recorded simultaneously and two electrophysiological indices of error monitoring, the error-related negativity (ERN) and error positivity (Pe) were measured. Amplitudes of each ERP were compared between diagnostic groups (CTRL, ADHD), medication days (Off MPH, On MPH) and motivational conditions (baseline – low incentive, reward, response cost).

**Results** Error rates were lower in the reward and response cost conditions compared with baseline across diagnostic groups and medication days. ERN and Pe amplitudes were significantly reduced in ADHD compared with CTRL, and were significantly enhanced by MPH. Incentives significantly increased ERN and Pe amplitudes in the ADHD group but had no effect in CTRL. The effects of incentives did not interact with the effects of MPH on either ERP. Effect sizes were computed and revealed larger effects of MPH than incentives on ERN and Pe amplitudes.

**Conclusions** The findings reveal independent effects of motivational incentives and MPH on two electrophysiological markers of error monitoring in children with ADHD, suggesting that each may be important tools for enhancing or restoring cognitive control in these children.

## Introduction

Children with attention deficit hyperactivity disorder (ADHD) are characterised by developmentally inappropriate levels of hyperactivity, impulsivity and/or inattention. Although early research posited ‘weak’ inhibitory control as the single factor underlying cognitive deficits in ADHD (Barkley, 1997), evidence of increased omission errors, greater reaction time (RT) variability (Kuntsi & Klein, 2012) and reduced posterror slowing (Yordanova et al., 2011) suggests that children with ADHD have a more generalised impairment in monitoring and regulating responses, not only response inhibition. However, the precise nature of these impairments in ADHD and the underlying causal processes remain to be determined.

To optimise performance in cognitive control tasks, it is essential to monitor one’s actions and adjust behaviour when necessary. Two electrophysiological markers thought to index these functions are the error-related negativity (ERN), a response-locked fronto-central event-related potential (ERP) peaks around 100 ms after a response; and the error positivity (Pe); a centro-parietal ERP with a latency of around 300 ms postresponse. The ERN and Pe follow different developmental trajectories, the ERN tending to increase in amplitude over development, whilst the Pe remains stable (Davies, Segalowitz & Gavin, 2004; Wiersema, van der Meere & Roeyers, 2007). Anatomically, the Pe has been localised to rostral ACC (Van Veen & Carter, 2002b) and posterior cingulate-precuneus (O’Connell et al., 2007) and the ERN to a more caudal region of ACC (Van Veen & Carter, 2002b). Functionally, the Pe is dependent upon error awareness (Nieuwenhuis, Ridderinkhof, Blom, Band & Kok, 2001), related to EEG measures of cortical arousal (Hajcak, McDonald & Simons, 2003; O’Connell et al., 2007) and may reflect the motivational evaluation of an error, or orienting of attention (Ullsperger, Harsay, Wessel & Ridderinkhof, 2010) as a precursor to performance adjustments (Overbeek, Nieuwenhuis & Ridderinkhof, 2005). Conversely, the ERN is not dependent on error awareness (Endrass, Reuter & Kathmann, 2007; Nieuwenhuis et al., 2001; O’Connell et al., 2007) and may index automatic detection of a mismatch between two competing responses (van Veen & Carter, 2002a) or an outcome that is worse than intended (Holroyd & Coles, 2002).

In ADHD, there is evidence of reduced ERN (Albrecht et al., 2008; Liotti, Pliszka, Perez, Kothmann & Woldorff, 2005; van Meel, Heslenfeld, Oosterlaan & Sergeant, 2007) and Pe (Groom, Cahill, Bates, Jackson, Calton, Liddle, et al. 2010; Jonkman, van Melis, Kemner & Markus, 2007; Shen, Tsai & Duann, 2011; Wiersema, van der Meere & Roeyers, 2005, 2009) amplitudes although there is inconsistency between studies as to which marker is affected (Shiels & Hawk, 2010). This could reflect pathophysiological heterogeneity in the ADHD population (Nigg, Willcutt, Doyle & Sonuga-Barke, 2005) but might also arise from differences in task design and performance given that error rate and RT are known to influence ERN amplitude (Hajcak et al., 2003). Differences in levels of motivation across experimental paradigms may also play a role because recent models of ADHD have proposed that higher level cognitive deficits may be partly attributable to problems with the regulation of arousal and motivational state (Sergeant, 2000) or reward processing (Tripp & Wickens, 2008). In healthy adults, the ERN responds to manipulations designed to modify the motivational salience of errors (Hajcak, Holroyd, Moser & Simons, 2005; Potts, 2011), raising the question of whether enhancing the motivational significance of errors can restore electrophysiological markers of error monitoring to typical levels in ADHD. If so, this could inform the development of behavioural strategies for optimising cognition in this population. Previously we reported significant effects of motivational incentives on amplitudes of two stimulus-locked ERPs, the N2 and P3, in the same groups described here (Groom, Scerif, Liddle, Batty, Liddle, Roberts et al., 2010). The incentives did not fully ‘normalise’ the N2 and P3 of the ADHD group compared with typically developing children raising the question of whether motivational incentives can be more effective when targeted at other parts of the cognitive control system, such as error monitoring.

To address these questions, we compared ERN and Pe amplitudes in children with and without ADHD during a go/no-go task. Children performed the task under three different motivational conditions in which the points awarded/deducted for accuracy on no-go trials relative to speed on go trials were manipulated between conditions. In a baseline (low incentive) condition the points awarded were equal. In two high-incentive conditions, the number of points awarded for successfully inhibiting the response on no-go trials (Reward condition) or deducted for errors on no-go trials (Response Cost condition) were greater than the points awarded for timely responses on go trials. We predicted that ERN and Pe amplitudes would be significantly greater in the high-incentive conditions when the motivation to exert inhibitory control on no-go trials and, consequently, the salience of errors, was greatest. We also examined interactions between the effects of motivational incentives and the presence/absence of ADHD diagnosis, hypothesising that if incentives ameliorate an underlying deficit in error monitoring in children with ADHD, this would be reflected in an interaction between diagnostic group and motivational condition, arising from a greater effect of incentives in the ADHD group than controls.

To investigate the effects of the indirect dopamine agonist methylphenidate (MPH) on error monitoring in ADHD, the children completed the task once on and once off their usual dose of stimulant medication. Based on evidence that dopamine agonists enhance error monitoring in healthy adults (de Bruijn, Hulstijn, Verkes, Ruigt & Sabbe, 2004; Hester et al., 2012) and ADHD (Jonkman et al., 2007), we predicted that amplitudes of the ERN and Pe would be enhanced by MPH, restoring them to similar levels as the control group. We also examined interactions between MPH and incentives in the ADHD group to determine whether incentives produced greater effects on ERN and/or Pe amplitude when combined with MPH.

## Method

### Participants

Participants were 28 children with DSM-IV ADHD (combined type) (27 males) aged 9–15 years and 28 typically developing controls matched on age (±6 months), gender, handedness [assessed using the Annett Handedness Scale (Annett, 1970)] and socioeconomic status (SES) [assessed using the Office for National Statistics SocioEconomic Classification system (Office for National Statistics, 2004)] (Table 1). Approval for the study was granted by the Nottingham Research Ethics Committee and the Research and Development Departments of Nottinghamshire Healthcare and Lincolnshire Partnerships NHS Trusts. Informed written consent with verbal assent was obtained from parents and children, respectively.

**Table 1:** Clinical and demographic characteristics of CTRL and ADHD groups

	CTRL (*n* = 28)		ADHD (*n* = 28)		Comparison	
	*M*	*SD*	*M*	*SD*	*t* (*df* = 54)	*p*
Age (years)	12.54	1.81	12.51	1.75	.07^a^	.946
Full-scale IQ	104.93	14.31	90.86	11.71	4.027	<.001
Diagnoses^c^						
AD/HD combined type	0		28		–	–
CD/ODD	0		21		–	–
Depression/anxiety	0		6		–	–
Conners (*T*-Score)						
DSM-Hyperactive	43.64	3.27	84.96	7.23	25.37^a^	<.001
DSM-Inattentive	43.65	3.22	73.50	7.96	18.36^a^	<.001
DSM-Total	44.25	3.32	81.61	7.69	23.62^a^	<.001
Oppositional	47.07	6.49	82.79	7.88	18.86^a^	<.001
DuPaul (ADHD-RS) (*T*-Score)						
Off meds	–	–	70.08	4.24	2.92^b^	.004
On meds	–	–	64.31	10.51		
Order of testing						
Off meds first	13		13			
On meds first	15		15			

CTRL, Control group; Conners, Conners Long Rating Scale – Teacher version; DSM, diagnostic and statistical manual of mental health disorders.

a Independent-samples *t*-test comparing CTRL and ADHD groups.

b Paired-samples *t*-test comparing off and on meds scores in the ADHD group; this analysis was conducted on 26 participants who completed both the off and on meds sessions.

c Frequency of diagnoses in each group. These were assessed using the Development and Well-Being Assessment (DAWBA) and calculated according to ICD10 and DSM-IV criteria. Groups were not compared statistically as diagnoses were absent from the control group.

Details of recruitment have been previously described (Groom, Scerif, et al., 2010); briefly, a consensus diagnostic conference (CDC) (involving CH and another experienced child & adolescent psychiatrist) was held for each case to determine study eligibility. The child’s medical notes were reviewed in conjunction with information from the following sources: Development and Well Being Assessment (DAWBA) (Goodman, Ford, Richards, Gatward & Meltzer, 2000); Strengths and Difficulties Questionnaire (SDQ) (Goodman, 1997); Conners Long Rating Scale-revised (Conners, 1997); Social Communication Questionnaire (SCQ) (Rutter, Bailey & Lord, 2003). Only right-handed children with a confirmed diagnosis of ADHD-combined type according to the CDC (corresponding to ICD-10 hyperkinetic disorder) and an established clinical response to MPH were included. Exclusion criteria for all participants were as follows: diagnosis of tic disorder, pervasive developmental disorder, learning disability [IQ <70, assessed with Wechsler Abbreviated Scale of Intelligence (Wechsler, 1999)] or neurological disorder.

Right-handed children with no known psychiatric diagnosis were identified from local schools and matched pairwise to a member of the ADHD group on age, gender and SES. Exclusion criteria were the same as those listed for the ADHD group. In addition, any participants with characteristics indicative of ADHD (score >5 on the hyperactivity subscale of the SDQ and/or *T* scores ≥60 on the Conners ADHD index) were excluded.

Diagnosis of anxiety, depression, oppositional defiant disorder (ODD) and conduct disorder (CD) did not warrant exclusion from either group.

### Task

A visual go/no-go task was programmed using E-Prime (version 1.1, Psychology Software Tools, Inc., Sharpsburg, PA, USA). Stimuli were presented centrally on a 17-inch colour monitor positioned approximately 57 cm in front of participants. Interstimulus interval was randomly jittered between 2.8 and 3.8 s; stimulus duration was 100 ms. Participants were instructed to fixate on a central point and press a response button (Cedrus Superlab button box) each time a frequent ‘go’ stimulus appeared and to refrain from responding to an infrequent ‘no-go’ stimulus. There were 600 trials with 150 (25%) no-go trials, presented in three motivational conditions (150 trials each), in the context of a ‘space’ theme; go stimuli were green aliens measuring 43 mm height by 40 mm width; no-go stimuli were black aliens of the same size. A time limit (‘RT cap’) was imposed on go trials: participants lost one point for slow or missed responses and gained one point for timely responses. Visual feedback was given 1000 ms poststimulus on slow or missed go trials.

To minimise between-subject variance in error rates the RT cap was dynamically altered by a tracking algorithm. An initial practice session comprising 20 Go trials used a stair-case procedure to identify the shortest time within which each participant could respond. This value became the lower bound of the tracking algorithm during the main experiment. The initial value of the tracking algorithm for each motivational condition was set at this value plus 200 ms. The cap then adjusted dynamically based on performance on no-go trials; following errors, the cap increased by 25 ms to improve the chance of success on the next no-go trial, whilst each successfully inhibited response resulted in a 25 ms decrease. There was an upper limit of 900 ms.

### Motivational incentives

Participants performed the task in three motivational conditions: baseline (low incentive); reward; response cost. Points awarded and deducted for response speed on go trials remained the same in all conditions whereas those awarded for correct/error responses on no-go trials were manipulated between conditions. In the baseline condition, participants gained one point for each successfully withheld response on a no-go trial, and lost one point for each commission error. In the reward condition, five points were given for each correctly withheld response with no penalty for errors and in the response cost condition, five credits were deducted for each error with no reward for successful inhibition. A visual stimulus was displayed in the top right corner of the monitor to remind participants which motivational block they were performing.

Stimuli were presented in five sets of three blocks (15 blocks in total), with each motivational condition randomly presented once within each set. Visual feedback at the end of each block summarised the numbers of points lost and gained. At the end of each set of three blocks, instructions emphasised either the need to withhold responses to the no-go stimulus or make timely responses to the go stimulus, depending upon whether participants achieved 50% successful inhibitions during that set.

### Procedure

Participants attended on two separate days, each included one EEG and one fMRI session [fMRI results are reported in (Liddle et al., 2011)]. The order of EEG and fMRI testing was counterbalanced and held constant across these days. Participants with ADHD were tested once off (medication withdrawal period of 48 hr) and once on their usual optimal medication dose (order counterbalanced). Group mean MPH dose was 1.11 (*SD* = 0.42) mg/kg/day. Symptoms were assessed on each day using the DSM-IV Rating scale (DuPaul, Power, Anastopoulos & Reid, 1998). Controls were never medicated but were tested twice to control for practice and boredom effects. Control (CTRL) and ADHD groups were pairwise-matched on sociodemographic variables and on the order in which each pair completed EEG and fMRI testing.

### Electrophysiological data recording and analysis

Data were recorded with a 256 Hz sampling rate between. 16 and 100 Hz using a Biosemi Active II system (Biosemi, Amsterdam, Netherlands) with 128-channel montage (including the standard 10–20 coordinates) of silver/silver-chloride (Ag/AgCl) electrodes. Additional electrodes were placed at the inner-orbital ridge and the outer canthi of each eye to record eye movements. During data collection, voltage signals were referenced to an electrode left of Cz.

Analysis was performed using Brain Vision Analyzer (BVA), version 2.0 (Brain Products, Gilching, Germany). After removal of persistently noisy/flat channels, data were re-referenced to the average of all electrodes and filtered with 0.5 Hz high-pass and 30 Hz low-pass zero-phase-shift Butterworth filters with slopes of 24 decibels/octave. Ocular artefacts were corrected using a linear regression method (Gratton, Coles & Donchin, 1983). As the primary focus of this analysis was error processing, no-go error trials (trials with a button-press occurring within 900 ms of a no-go stimulus) were selected and other trials were discarded. There were insufficient errors on go trials (slow or missed responses) to allow a comparison of differences between go and no-go errors. The data were segmented into 2000 ms epochs centred on button-press response. Trials with activity exceeding ±100 μV at any point within the epoch, or less than 2.5 μV for more than 500 ms were marked as artefacts and excluded. Baseline correction was performed using a 200 ms reference period commencing 800 ms prior to the response. This window was chosen to minimise overlap with stimulus-locked ERPs. Data were then averaged across trials within each motivational condition per subject and session. The ERN was defined as peak voltage in a peri-response window of −10 ms to 100 ms. The Pe was computed as mean amplitude within a 200–400 ms time window postresponse. These time windows were based on typical time windows in previous studies (Albrecht et al., 2008; Falkenstein, Hoormann, Christ & Hohnsbein, 2000; Wiersema et al., 2005) and visual inspection of grand average waveforms. The ERN was measured at FCz and the Pe at CPz, the sites of maximum amplitude across groups, medication sessions and motivational conditions (see Figures 2B and 3B).

To improve measurement of peak ERN amplitude, the data were filtered between 2 and 15 Hz prior to segmenting into epochs (the Pe was measured without this additional filtering step). Previous research has shown that the ERN is predominantly composed of theta (4–7 Hz) and alpha (8–13 Hz) power (Luu, Tucker & Makeig, 2004); these filters will therefore preserve the activity of interest, while removing the influence of overlapping low-and high-frequency activity, which could confound measurement of ERN amplitudes.

### Statistical analysis

A paired analysis was conducted in which the off and on medication days of each ADHD participant were paired with the equivalent day (1 or 2) of their matched control. Of 28 CTRL-ADHD pairs recruited to the study, two ADHD participants did not complete the EEG session when un-medicated and one CTRL did not complete testing on the day equivalent to the on medication day of their matched ADHD participant, leaving 25 fully paired datasets for analysis.

Repeated measures ANOVAs were conducted on error rates and on the amplitudes of each ERP (ERN, Pe). Each ANOVA consisted of three within-subjects factors: Diagnosis (CTRL, ADHD), Day (Off MPH, On MPH) and Motivation (Baseline, Reward; Response Cost). This fully paired design capitalised on the careful pairwise matching of the CTRL and ADHD groups on age, gender and SES. Off MPH and On MPH refer to the un-medicated and medicated testing days of participants in the ADHD group, respectively, and to the equivalent day (1 or 2) in their matched CTRL. Interactions significant at *p* <.05 were analysed further. Significant effects of Motivation were analysed with planned orthogonal contrasts comparing, first, the Baseline condition with the mean of the two motivational incentive conditions (Reward and Response Cost), and second the Reward and Response Cost conditions. Effect sizes (partial eta squared; *η*_p_^2^) are reported.

Two participants (one CTRL, one ADHD) had too few trials for ERP averaging (<20) in one or more motivational conditions and were excluded from analysis. For the ERN, one CTRL participant was a multivariate outlier (standardised residual >3 on at least two variables). The matched partners of these excluded participants were also removed from all analyses involving both groups, but not from analyses involving single groups or days.

The ERP analyses were re-run to determine whether main effects of Diagnosis were influenced by group differences in IQ. IQ was not a significant predictor of either ERN or Pe amplitude and did not alter the Diagnosis effects. The analyses were also re-run after excluding children with comorbid anxiety (*n* = 4) or depressive (*n* = 1) disorder as these might be expected to influence ERN amplitude. The pattern of significant effects was unchanged. Finally, to determine whether the high prevalence of CD/ODD diagnosis in the ADHD group (*n* = 21) influenced the ERP findings, Pearson’s correlation coefficients were computed between CD/ODD symptom ratings from the teacher Conners scale and ERP amplitudes. All were nonsignificant (*p* >.1). The results are therefore reported without adjustment for IQ and comorbidities.

## Results

### Performance

**Figure 1:**
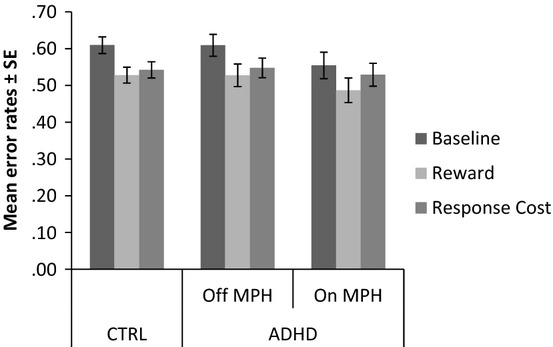
Error rates in the CTRL group and the ADHD group Off MPH and On MPH days, by motivational condition. The CTRL group data are collapsed across both days

Analysis of error rates confirmed that the tracking algorithm was effective in minimising differences between subjects and testing sessions (Figure 1). There was no effect of Diagnosis, Day or a Diagnosis-by-Day interaction (all *p* < 1). A main effect of Motivation (*F*(2, 42) = 18.76, *p* <.001) revealed significantly lower error rates in the Reward and Response Cost conditions compared with Baseline (*F*(1, 21) = 32.78, *p* <.001), indicating that participants adjusted their performance according to motivational incentives. Motivation did not interact with Diagnosis or Day (*p* >.1).

### ERN amplitude

There was no significant main effect of Diagnosis (*p* >.1), but a significant Diagnosis-by-Day interaction (*F*(1, 21) = 10.03, *p* =.005, *η*_p_^2^ =.32). There was also a significant Diagnosis-by-Motivation interaction (*F*(2, 42) = 4.24, *p* =.02, *η*_p_^2^ =.17).

**Figure 2:**
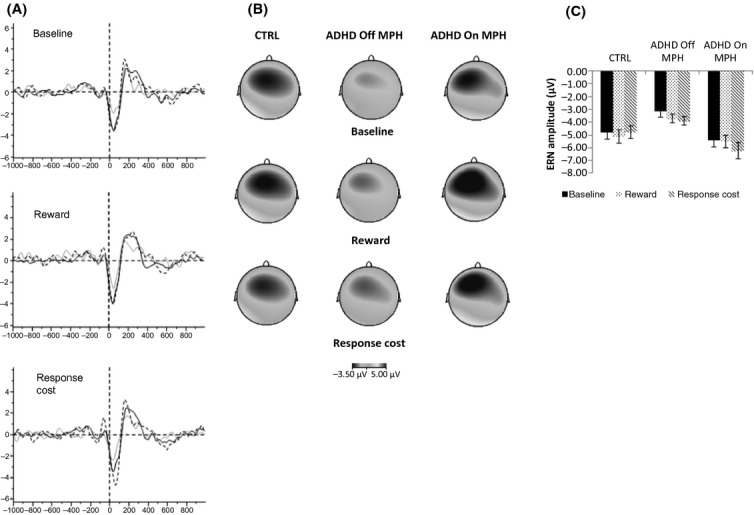
(A) ERP waveforms at FCz for each motivational condition in the CTRL group (solid black line), ADHD Off MPH (solid grey line) and ADHD On MPH (dashed line). Each plot shows amplitude in microvolts on the *y* axis and time in milliseconds on the *x*-axis. (B) Topographic plots showing the distribution of amplitudes on the scalp in microvolts at peak ERN amplitude for each group/day and motivational condition. (C) Mean amplitude in each motivational condition for the CTRL group, ADHD Off MPH and On MPH. The CTRL group data are collapsed across both days in all plots

To clarify the effects indicated by the Diagnosis*Day interaction, three repeated-measures ANOVAs were conducted. First, the two diagnostic groups (ADHD and CTRL) were compared using data from the Off MPH day. This yielded a significant effect of Diagnosis (*F*(1,21) = 5.49, *p* =.03, *η*_p_^2^ =.21) with smaller amplitude in the ADHD group. Next, the two groups were compared using data from the On MPH day. No significant difference was found (*p* >.1). Finally, an ANOVA was performed on data from the ADHD group only and revealed a main effect of medication Day (*F*(1,24) = 24.35, *p* <.001, *η*_p_^2^ =.5) with greater ERN amplitude On than Off MPH, shown in Figure 2A.

To clarify the effects indicated by the Diagnosis-by-Motivation interaction, repeated-measure ANOVAs were performed on each group separately. In the CTRL group, there was no effect of Motivation (*p* >.1). In the ADHD group, there was a main effect of Motivation (*F*(2,48) = 3.52, *p* =.04, *η*_p_^2^ =.13), which did not interact with medication Day (*p* >.1) (see Figure 2C)^1^ The planned contrast revealed significantly greater amplitude in the motivational conditions (Reward and Response Cost) compared with Baseline (*F*(1,24) = 4.54, *p* =.04, *η*_p_^2^ =.16), and a trend for greater amplitude for Response Cost than Reward (*F*(1,24) = 2.49, *p* =.1, *η*_p_^2^ =.09). The pattern of effects was further explored using a polynomial contrast to determine whether there was a significant relationship between ERN amplitude and the magnitude of the penalty for errors on no-go trials. The Baseline condition yielded the smallest penalty (loss of one point), followed by slightly greater penalty in the Reward condition (missed opportunity to gain five points), and the greatest penalty in the Response Cost condition (loss of five points earned on previous trials). The linear term was significant (*F*(1,22) = 8.23, *p* =.008, *η*_p_^2^ =.27).

### Pe amplitude

There was no main effect of Diagnosis but a Diagnosis-by-Day interaction (*F*(1, 22) = 6.17, *p* =.02, *η*_p_^2^ =.22). There was also a main effect of Motivation (*F*(2, 44) = 4.27, *p* =.02, *η*_p_^2^ =.16) that interacted significantly with Diagnosis (*F*(2, 44) = 3.99, *p* =.03, *η*_p_^2^ =.15).

**Figure 3:**
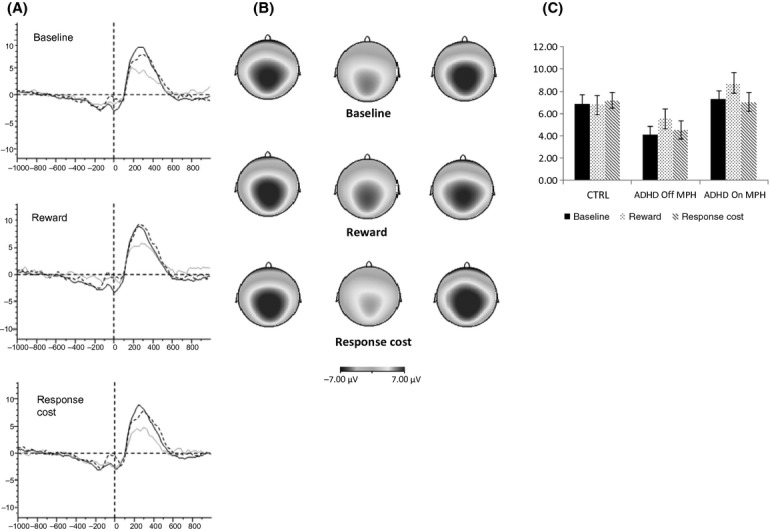
(A) ERP waveforms at CPz for each motivational condition in the CTRL group (solid black line), ADHD Off MPH (solid grey line) and ADHD On MPH (dashed line). Each plot shows amplitude in microvolts on the *y* axis and time in milliseconds on the *x*-axis. (B) Topographic plots showing the distribution of amplitudes on the scalp in microvolts at peak Pe amplitude for each group/day and motivational condition. (C) Mean amplitude in each motivational condition for the CTRL group, ADHD Off MPH and On MPH. The CTRL group data are collapsed across both days in all plots

Follow-up analyses were performed as described above. When groups were compared on the Off MPH day, the effect of Diagnosis approached significance (*F*(1,22) = 4.13, *p* =.05) with greater amplitude for CTRL. There was no significant group difference for the On MPH day (*p* >.1). For the ADHD group, there was a main effect of medication Day (*F*(1,24) = 35.29, *p* <.001, *η*_p_^2^ =.60) with greater amplitude On MPH than Off MPH (Figure 3A).

When effects of Motivation were tested in the two groups separately, there was a significant main effect of Motivation in the ADHD group (*F*(2,48) = 8.50, *p* =.001, *η*_p_^2^ =.26), but no Motivation-by-Day interaction (*p* >.1). In the CTRL group, as with the ERN analysis, there was no significant effect of Motivation (*p* >.1). The planned contrasts in the ADHD group revealed significantly greater Pe amplitude for Reward than Response Cost (*F*(1,24) = 9.96, *p* =.004, *η*_p_^2^ =.29)^2^. As the effects of Motivation did not show the predicted pattern (Reward and Response Cost greater than Baseline), further pairwise *t*-tests were conducted. These revealed significantly greater amplitude in the Reward condition than the Response Cost (*p* =.02) and Baseline (*p* =.02) conditions (Bonferroni corrected), which did not differ from one another (*p* >.1) (Figure 3C).

## Discussion

This study investigated the effects of motivational incentives and stimulant medication on electrophysiological correlates of performance monitoring in children with ADHD. ERN and Pe amplitudes in the ADHD group were reduced when withdrawn from MPH compared with pairwise-matched typically developing controls. The amplitudes of both ERPs were significantly increased by MPH and by motivational incentives in the ADHD group, and these effects did not interact.

The finding of reduced ERN and Pe amplitudes in the ADHD group is consistent with previous research (Albrecht et al., 2008; Groom, Cahill, et al., 2010; Jonkman et al., 2007; Liotti et al., 2005; van Meel et al., 2007; Shen et al., 2011; Wiersema et al., 2005, 2009), although this is the first study to identify abnormalities in both ERPs in the same sample suggesting that when between-subject variability in error rates is removed, early error detection (ERN) and later evaluation of the error (Pe) are both atypical in ADHD. We also identified two methods for enhancing ERN and Pe amplitudes in ADHD. First, stimulant medication produced a highly significant effect on the amplitudes of both ERPs. Although compelling, one of the drawbacks of stimulant medication is that the positive effects of the drug may not persist after withdrawal (Swanson, Baler & Volkow, 2011). Alternative interventions with longer lasting effects are therefore of potential utility. Here, we provide novel evidence of the significant effects of motivational incentives on the ERN and Pe in ADHD, indicating that deficits in higher cognitive functions in this population can be reduced by enhancing the motivational significance of task-relevant stimuli, supporting the proposition that deficits in cognitive control are at least partly attributable to problems with the regulation of arousal and motivational state (Sergeant, 2000) or reward processing (Tripp & Wickens, 2008). Further study is needed to determine whether the positive effects of motivational incentives are sustained over the longer term. We found no evidence that MPH enhanced the efficacy of motivational incentives suggesting that incentives can improve performance monitoring in ADHD without the need for medication, although the effect sizes for incentives were smaller than those for MPH. We previously reported independent effects of MPH and incentives on stimulus-locked ERPs (Groom, Scerif, et al., 2010) and the default mode network (DMN) (Liddle et al., 2011) in this sample. Integrating these findings, incentives and MPH independently improve several stages of cognitive control in ADHD: attention to a task-relevant stimuli (DMN deactivation), inhibitory control (N2 amplitude), error detection (ERN amplitude), stimulus (P3 amplitude) and response (Pe amplitude) evaluation. By challenging these key stages of the cognitive control system, the findings help clarify the effects of MPH and incentives on cognition in ADHD (Swanson et al., 2011).

The effects of motivation in the ADHD group differed between the ERN and Pe. ERN amplitude followed a linear pattern related to the size of the penalty for error commission, indicating that children’s error detection processes were influenced to a greater extent by the possibility of losing credits gained on previous trials than by a missed opportunity to gain further credits. Conversely, Pe amplitude, and thus response evaluation, was significantly greater in the reward condition than either the response cost or baseline conditions. Previous studies have reported insensitivity to penalties in children with ADHD compared with oversensitivity to loss of anticipated rewards, although others have found the opposite pattern [reviewed in (Luman, Oosterlaan & Sergeant, 2005)]. Further study to elucidate the neural systems underlying the ERN and Pe and how these are modulated by motivational incentives in healthy individuals as well as those with ADHD, is needed.

In one influential theory of reinforcement learning (Holroyd & Coles, 2002), the ERN is conceptualised as a phasic interruption in tonic mesolimbic dopamine that acts as a signal to prefrontal brain regions that an outcome is worse than expected. Although this study did not manipulate the availability of DA selectively, the increase in ERN amplitude in the ADHD group when tested on MPH or when provided with motivational incentives lends some support to a role for DA in the modulation of the ERN, and to models of ADHD in which the features of the disorder are attributable to dysfunction in either the tonic or phasic release of midbrain DA [reviewed in (Cockburn & Holroyd, 2010)]. This does not preclude the importance of other neurotransmitters and their interactions with DA in explaining the effects of MPH and incentives. In particular, noradrenaline is modulated by MPH (Del Campo, Chamberlain, Sahakian & Robbins, 2011) and incentives (Sara, 2009).

One important difference between the findings of the stimulus-locked (Groom, Scerif, et al., 2010) and response-locked analyses is that in the control group incentives increased N2 and P3 amplitudes, but not ERN and Pe amplitudes. One other study (Torpey, Hajcak & Klein, 2009) found no effect of incentives on ERN amplitude in typically developing children aged 5–7 years. Perhaps greater incentives or a more difficult cognitive task are required for a measurable effect on error detection to occur in typically developing children. Alternatively, the effects of incentives in typical development may be specific to stimulus-processing rather than response monitoring. Further study is needed to probe more fully factors that influence the efficacy of incentives in typically developing children as well as those with ADHD, including the magnitude and frequency of reinforcers, symptom severity in ADHD and the components of the cognitive control system at which the strongest effects occur. Careful exploration of these parameters could facilitate the development of behavioural interventions that enhance the neural processes underlying impaired cognitive control in ADHD.

**Key points**

1. It is known that performance monitoring during challenging cognitive tasks is impaired in children with ADHD. This is reflected in reduced amplitude of two electrophysiological markers of error monitoring.
2. We found that stimulant medication (methylphenidate), when administered at the usual and therefore clinically optimal dose of children with ADHD, enhances the amplitudes of these markers. We also found that performance-based motivational incentives enhanced amplitudes, although the effect size for incentives was smaller than for medication.
3. The clinical implications of this study are that medication and incentives are effective in ameliorating deficits in this aspect of cognition in children with ADHD.

## Notes

1 These effects were replicated when modelling Diagnosis as a between-groups factor yielding an effect size of.13 for motivational effects in the ADHD group compared with.07 in the CRTL group, reflected in a Diagnosis*Motivation interaction (*F*(2, 98) = 5.45, *p* =.006, *η*_p_^2^ =.1).

2 The pattern of effects was replicated with Diagnosis modelled as a between-groups factor. There was a Diagnosis*Motivation interaction (*F*(2, 98) = 3.65, *p* =.03, *η*_p_^2^ =.07) arising from a significant effect of Motivation in the ADHD group (*F*(2, 48) = 6.24, *p* =.004, *η*_p_^2^ =.21) but not the CTRL group (*p* >.1).
